# Hospital Care and the Conception of Death in the Hospitaller Order of Saint John of God in Sixteenth‐ and Seventeenth‐Century Spain

**DOI:** 10.1111/nin.70042

**Published:** 2025-07-06

**Authors:** Aarón Muñoz Devesa, Juan Ignacio Rico Becerra

**Affiliations:** ^1^ Faculty of Social and Health Sciences University of Murcia Lorca Region of Murcia Spain

**Keywords:** Christian anthropology, early modern Spain, end‐of‐life care, ethical care, ethical nursing practice, funeral rites, history of nursing, hospitaller care, spiritual care, spirituality in health care

## Abstract

This article explores the hospital care provided by the Hospitaller Order of Saint John of God in sixteenth‐ and seventeenth‐century Spain, with particular emphasis on its conception of end‐of‐life care. Rooted in a context deeply shaped by Christian spirituality, the Order developed a holistic model grounded in charity, justice, and profound respect for the dignity of the sick. Through a critical analysis of normative and doctrinal sources, the study reconstructs caregiving practices surrounding dying and post‐mortem care, highlighting their institutionalization and the Order's hopeful understanding of death as a transition to eternal life. These practices were codified in detailed regulations concerning the accompaniment of the dying, the administration of sacraments, care for the deceased body, and the offering of suffrages. Employing a hermeneutic lens, the article interprets these practices within their original theological and cultural horizon, consciously avoiding the imposition of contemporary frameworks. Simultaneously, it argues that this historical legacy of ethical and spiritual care can enrich current reflections on death, nursing practice, and the role of institutions in safeguarding human dignity at the end of life.

## 1 Introduction

The Hospitaller tradition of death and end‐of‐life care developed in Spain during the sixteenth and seventeenth centuries—a period marked by profound economic, demographic, religious, and cultural transformations (Ariès and Duby 2000a; Elías 1987; Fernández Álvarez 2015; Foucault 1990; Martín 2004). While the sixteenth century witnessed imperial expansion and population growth, it remained a rigidly stratified society in which the majority of people lived in precarious conditions (Fernández Álvarez 2006; Sanz and Camañes 2012). In the seventeenth century, these difficulties intensified due to a succession of economic crises, widespread epidemics, the expulsion of the Moriscos—Muslims forcibly converted to Christianity who remained under suspicion of heresy—and significant waves of emigration. These circumstances created a climate of uncertainty and fostered a renewed search for spiritual consolation and care for human suffering (Fernández Álvarez 1984; Iáñez 1989; Ventosa 1984).

Within this broader context—despite the presence of critical voices—the Catholic Church maintained a dominant role in shaping everyday life, with a strong emphasis on spiritual preparation for death (Cabañas 2017; Díaz Villanueva and Garín 2022). Health care, poorly regulated and institutionally fragmented, largely depended on religious orders that assumed responsibility for hospital care among the most vulnerable populations (Gijón Jiménez 2018; López Terrada and Schmitz 2018). Hospitaller orders were Catholic religious institutions devoted to the care of the sick, the poor, and the dying, operating hospitals across Europe from the medieval to the early modern period (Conejo da Pena and Bridgewater Mateu 2023). Their assistance combined spiritual guidance, physical caregiving, and moral discipline, all within a theologically grounded framework. Hospitals and foundling homes serving the destitute and chronically ill registered high mortality rates, often due to inadequate infrastructure and rudimentary medical practices (Ariès and Duby 2000b; Fernández Álvarez 2006; García Oro and Portela Silva 2000; Granjel 1980).

In response to these pressing needs, a number of Hospitaller orders emerged. Among them were the Camillians, who focused on patients with contagious diseases, and the Obregonians, pioneers in the professionalization of nursing care (Hernández Martín 1996; Siles and González 2009; Antonelli et al. 2015). The Hospitaller Order of Saint John of God arose from the personal initiative of João Cidade (later canonized as Saint John of God), a former soldier and laborer who, following a radical spiritual conversion, began caring for the sick and abandoned in Granada. Although he did not initially intend to found a religious congregation, the spiritual and social impact of his work led local authorities and the ecclesiastical hierarchy to support the formal establishment of an institution. His followers—among them Antón Martín and Pedro Velasco—soon systematized his practices into a cohesive framework of care. Within this evolving landscape, the Order distinguished itself by integrating medical care with spiritual accompaniment, developing a model centered on the dignity of the sick and their preparation for death. The originality of the Order lay not only in its holistic approach but also in the early institutionalization of detailed caregiving norms—an innovation that decisively shaped early modern conceptions of dying and spiritual care.

In this context, this article examines the end‐of‐life care practices of the Hospitaller Order of Saint John of God in sixteenth‐ and seventeenth‐century Spain. Drawing on normative and doctrinal sources—including constitutions, manuals, hagiographies, and letters—the study reconstructs the Order's conception of death and caregiving practices related to dying and post‐mortem care. Adopting a hermeneutic approach, it interprets these practices within their original theological and cultural context, deliberately avoiding presentist projections. Rather than retroactively applying modern assumptions to early modern caregiving, the study seeks to understand how these practices emerged as a coherent response to the vulnerabilities of illness, dying, and spiritual anxiety. At the same time, it argues that this historical legacy of structured, ethically grounded, and spiritually informed care offers critical insight into the ongoing quest to humanize end‐of‐life experiences in contemporary nursing.

This article does not seek to engage in theoretical historiographical debates but instead provides a document‐driven reconstruction in the field of cultural history, focusing on practices of care and death. By analyzing primary normative texts and ritual sources, it interprets the end‐of‐life practices of the Hospitaller Order of Saint John of God, contextualizing them within their theological and anthropological frameworks. In doing so, the study advances the cultural historiography of spiritually grounded caregiving while engaging with scholarship on Catholic death rituals and early modern religious caregiving traditions. Here, *historiography* is not used to indicate participation in a specific historical tradition but to describe a methodological orientation grounded in cultural analysis of caregiving practices.

## 2 Methodology

This study, situated within the field of nursing historiography, adopts a qualitative, interpretive approach grounded in hermeneutic methodology. This orientation enables a historically sensitive reconstruction of caregiving practices and symbolic representations, with particular attention to the internal coherence of normative and doctrinal sources. Rather than imposing predefined analytical frameworks, the hermeneutic process seeks to understand the meaning of practices and regulations within their original theological, institutional, and cultural context.

The primary sources analyzed in this study are summarized in Table 1. These documents, produced or used by the Hospitaller Order of Saint John of God between the sixteenth and seventeenth centuries, include constitutions, manuals, hagiographies, legal texts, and spiritual rules. They form the core corpus from which the interpretive categories of the present study were inductively derived.

**Table 1:** Primary sources analyzed in the study and their English translations.

	English Translation	Year
Regla y Constituciones para el Hospital de Juan de Dios de la ciudad de Granada	Rule and Constitutions for the Hospital of John of God in the City of Granada	1585
Constituciones de la Orden Hospitalaria de San Juan de Dios	Constitutions of the Hospitaller Order of Saint John of God	1587, 1611
Bula Licet ex debito (Pope Pius V)	Papal Bull *Licet ex debito*	1572
Manual de administración de sacramentos	Manual for the Administration of Sacraments	1666
Instrucción de novicios	Instruction for Novices	1668
Cartas de San Juan de Dios	Letters of Saint John of God	2006
Pleito entre los hermanos del hospital y los frailes del monasterio de San Jerónimo	Dispute between the Brothers of the Hospital and the Monks of San Jerónimo Monastery	2007
Proceso de beatificación de San Juan de Dios	Beatification Process of Saint John of God	2006
Historia de la vida y santas obras de San Juan de Dios	History of the Life and Holy Works of Saint John of God	1995
Regula Sancti Augustini	Rule of Saint Augustine	n.d.

*Note:* All translations from the original Spanish or Latin documents were carried out by the author. All sources were analyzed in their original language. No digital transcription tools or AI‐based translation systems were used, ensuring philological fidelity to the texts.

These documents were analyzed through a hermeneutic process that sought to recover their internal coherence, symbolic structures, and embedded values. Interpretive attention was given to their theological language, ritual prescriptions, ethical assumptions, and institutional concerns, allowing categories to emerge inductively from the texts themselves. Rather than applying predefined codes, the analytical approach was iterative and dialogical, allowing meanings to surface through repeated close readings and constant comparison between sources. Throughout the process, the study deliberately avoided imposing contemporary frameworks or retroactively modernizing the content. Nonetheless, the ethical and anthropological significance of these practices is acknowledged from a contextually grounded, non‐anachronistic perspective.

To further support the analysis, secondary literature on the history of nursing, hospital spirituality, and early modern representations of death was consulted. The contributions of scholars such as Martínez Gil (1993, 2006), Hernández Martín (1996), Siles and González (2009), Ventosa (2012), and Amezcua (2019) provide a rigorous and up‐to‐date historiographical framework.

Following a hermeneutic approach, a thematic‐categorical analysis was conducted to identify four major interpretive axes that structure the study. These axes emerged through repeated readings of the primary documents and comparative cross‐referencing with secondary sources. The first axis concerns the ethical principles that guided the mission of the Order of Saint John of God, particularly its emphasis on charity, justice, and hospitality. The second addresses the Order's theological conception of death as a transition to eternal life and a moment of spiritual judgment. The third focuses on the institutionalized practices of end‐of‐life care, including spiritual accompaniment, administration of sacraments, and bodily care during agony. Finally, the fourth axis examines post‐mortem rituals and remembrance practices, including care of the corpse, liturgical suffrages, and the preservation of memory.

Each thematic category was developed through an iterative process of critical reading, coding, and constant comparison between sources. The primary documents were not interpreted in isolation, but were cross‐referenced with a carefully selected corpus of secondary literature that contextualizes early modern religious, medical, and caregiving practices. To enhance the methodological rigor of the analysis, a strategy of documentary triangulation was employed, whereby the normative texts of the Order were contrasted with established historiographical interpretations and broader theoretical frameworks on death and care (Ariès & Duby 2000; Elías 1987; Foucault 2004; Fernández Álvarez 2006; Haindl Ugarte 2013). This allowed the study to verify the internal consistency of its findings while situating them within the scholarly discourse on spiritual care and the anthropology of death (Sánchez‐Martínez 2007).

It is important to note that terms such as *spirituality*, *humanization*, or *dignity* do not appear explicitly in the original documents. However, these notions emerge implicitly through the Order's regulatory language, ritual structures, and caregiving discourses, which consistently emphasize moral responsibility, compassionate presence, and the spiritual destiny of the sick. Their inclusion in the analysis is the result of a critical, hermeneutically grounded reading, in which contemporary ethical and anthropological concepts are used not as interpretive impositions but as heuristic devices to illuminate the ethical textures of historical practices.

This interpretive strategy does not imply a retroactive attribution of modern categories, but a disciplined reconstruction of how ethical and anthropological values were expressed through the language and rituals of the time.

This approach allows the study to articulate a meaningful bridge between past and present, without compromising historical fidelity.

## 3 Findings and Interpretations

This section explores the hospital care practices of the Hospitaller Order of Saint John of God and its theological conception of death in sixteenth‐ and seventeenth‐century Spain. The analysis is structured according to the four thematic categories previously outlined: ethical principles, the meaning of death, end‐of‐life practices, and post‐mortem care. Each axis is examined through a hermeneutic reading of normative texts, ritual regulations, and doctrinal sources, with attention to their theological coherence, institutional objectives, and anthropological implications. This approach aims to reconstruct how the Order configured a spiritually grounded model of care that may offer critical insights for contemporary nursing ethics and end‐of‐life practice.

### 3.1 The Hospitaller Order of Saint John of God and Its Ethical Principles

Saint John of God (1495–1550) played a pivotal role in the reform and development of hospital care in sixteenth‐century Spain. His first‐hand experiences in the hospitals of Granada—marked by poor hygiene, insufficient medical attention, and widespread neglect of the most vulnerable—profoundly shaped his conception of caregiving as a holistic and ethically grounded vocation.

In response to these deficiencies, he founded a hospital in Granada in 1537. His model of caregiving integrated curative treatments with broader measures aimed at enhancing patient well‐being, including improved hygiene, better spatial organization, and, above all, more humane and compassionate treatment. This integrative vision anticipated later conceptions of holistic care, as it explicitly addressed both the physical and spiritual needs of the sick (Castro 1995).

After his death in 1550, his disciples—most notably Antón Martín, Pedro Velasco, and Pedro Soriano—formalized his legacy through the foundation of the Hospitaller Order of Saint John of God. This process culminated in official recognition by the Catholic Church through the papal bull *Licet ex debito*, issued by Pope Pius V in 1572, which marked the beginning of a period of rapid institutional expansion and normative codification.

By the end of the seventeenth century, the Order operated numerous hospitals not only in Spain—in cities such as Granada, Seville, and Toledo—but also across Europe, including Italy, France, Poland, and Portugal. Its presence extended beyond the continent to the Americas (e.g., Cartagena de Indias and Mexico City), Asia (the Philippines), and Africa (Mozambique). This widespread diffusion attests not only to the acceptance of the Order's caregiving model but also to its capacity for institutional adaptation and normative coherence across diverse sociocultural contexts (Orden Hospitalaria de San Juan de Dios 2019, s.f.; Ventosa 2012).

Although the Hospitaller Order of Saint John of God was canonically male and governed by the spiritual norms of Augustinian religious life, its hospitals systematically included female patients and integrated women into caregiving structures. Access to care was granted to all regardless of sex, and women were received in dedicated wards, attended by female personnel such as the Madre Enfermera Mayor and her assistants, whose roles were strictly regulated in terms of competence, virtue, and religious modesty. The organization of female care was deliberately separated from male wards, and strict rules governed access, movement, and communication between the sexes. This structure, shaped by ecclesiastical norms and moral theology, ensured not only physical protection but spiritual safeguarding for both caregivers and patients. Moreover, the quality of care for women was expected to match that of men in all aspects—medical attention, nutrition, comfort, and ritual accompaniment. Beyond the institutional framework, the Order also extended specific forms of caritas toward women in vulnerable social positions—widows, orphans, women at risk of dishonor—while female benefactors played a central role in sustaining the hospital economy. As a whole, the system reveals a gendered ethic of care: one that was segregated in structure but aimed to be universal in mission, deeply informed by the theological imperatives of charity, modesty, and salvific dignity.

#### 3.1.1 Charity and the Hospitaller Vocation

From its inception, the Order emerged in response to urgent social and spiritual needs during a period of profound societal transformation (Martín 2004; Fernández Álvarez 1984). Charity was not merely a moral virtue but a foundational operational principle that structured the Order's caregiving ethos and institutional identity (Gijón Jiménez 2018; López Terrada and Schmitz 2018). As Saint John of God emphatically declared, “Always practise charity, for where there is no charity, there is no God” (Fernández de Viana y Vieites et al. 2006, 152).

Consequently, the care of the sick was conceived not as an occasional act of beneficence, but as a structured and permanent expression of Christian love and divine compassion. This orientation was formally codified in the Rule and Constitutions, which explicitly stated:

The principal purpose of this hospital is the care and comfort of the poor of Jesus Christ. We entrust the Brother Superior to be gentle, compassionate, and charitable with the poor, to be deeply moved by their afflictions, not to be annoyed or troubled by their demands, but rather to comfort and assist them with kind words and charitable deeds.

(Méndez de Salvatierra 1585, Title VII, Const. 9)

This virtue was to be actively cultivated through ritualized daily practices, as clearly articulated in the *Instruction for Novices*. There, caregiving is not described in abstract theological terms, but through concrete bodily actions and moral dispositions that structure everyday life:

Charity is shown in making the beds of the sick, feeding them, comforting them in their afflictions, treating their ailments, helping them to die well; in others, humility is demonstrated in sweeping the infirmaries and cleaning away all filth; in others, patient perseverance in searching for their food. In all, a steadfast love of God is enacted through service to the needy.

(Victoria 1668, chap. II)

Charity, therefore, encompassed both inner disposition and outward action. It shaped the ethos of hospitality, functioning not only as a form of social protection but also as a disciplinary pedagogy, aimed at instilling specific values and behaviors among the poor through structured care practices (Ariès 1999; Foucault 2004; León Vegas 2013).

Fundamentally, charity was inseparable from the hospitaller vocation, demanding unconditional self‐giving in both intention and practice. It was understood not only as a theological imperative but as an institutional mandate embodied in the daily ethics of care. The sick were to be treated with the same reverence one would offer to Christ himself: “What would you not do for Him? […] Understand, my brothers, that what you do in service of each poor person, you do for Christ Himself” (Introduction, Rule and Constitutions; Méndez de Salvatierra 1585).

#### 3.1.2 Justice and Universal Care

Justice was another core ethical principle for the Order. Saint John of God defined it as the imperative “to be just, giving each person what is rightfully theirs” (Third Letter to the Duchess of Sessa, Fernández de Viana y Vieites et al. 2006, 20). Within the Order's framework, justice functioned not only as a theological ideal but as a regulatory principle that ensured access to care without discrimination. This commitment was formally codified in the 1587 Constitution: “Let them receive with all love the poor and sick from all nations and with all illnesses, without distinction or exception of persons” (Méndez de Salvatierra 1587, Title I, Const. 15).

This mandate was further reiterated in the *Instruction for Novices*: “We are obliged to receive and care for all the sick who appeal to our charity, providing them with beds, sustenance, and necessary medicines, and attending to them personally—regardless of cost, fatigue, or risk” (Victoria 1668, chap. XXV, § IV).

In an era marked by widespread poverty and limited medical resources, the Order advanced a structured, normatively regulated, and inclusive model of care centered on the inherent dignity and diverse needs of each patient (García‐Villoslada 1979; Ventosa 1984, 2006; Fernández Álvarez 2015).

#### 3.1.3 Material Renunciation and Spiritual Devotion

The principle of material renunciation constituted a crucial element in the ethical framework of the Hospitaller Order. The brothers embraced voluntary poverty as a normative condition of religious life and as a means to dedicate themselves fully to the biopsychosocial and spiritual needs of the sick, particularly those nearing the end of life. This deliberate choice stood in stark contrast to the prevailing values of a stratified society, wherein honor and material wealth served as markers of social prestige. The Order, by contrast, promoted the disciplined use of material goods strictly oriented to spiritual purposes and caregiving duties.

Saint John of God articulated this vision with striking clarity: “Reflect on how death consumes and destroys all that this miserable world offers us, and how we leave with nothing but a piece of torn and poorly stitched linen” (Third Letter to the Duchess of Sessa, Fernández de Viana y Vieites et al. 2006, 123). This perspective framed material detachment not only as spiritual wisdom, but as a form of ethical preparation for caregiving: the brother's personal renunciation mirrored the existential fragility of the sick and reinforced the humility required to accompany them.

This worldview aligned with the Rule of Saint Augustine, which framed poverty as a pathway to spiritual perfection and service to others (Agustín de Hipona s.f). Within the Hospitaller Order, this principle was not merely symbolic, but institutionally enacted through norms, practices, and communal life. Through this ethical orientation, the Order actively challenged dominant social norms regarding honor, wealth, and status (Ariès 2005).

#### 3.1.4 Respect for the Dignity of the Sick

Although sixteenth‐century medicine had not yet conceptualized patient autonomy in modern terms, the Hospitaller Order of Saint John of God fostered a heightened sensitivity to the individual dignity of each patient. This concern was not limited to spiritual discourse but was institutionally expressed through caregiving norms and daily practices. Their approach resonated with the early modern emergence of the individual as a singular subject—a phenomenon described by Ariès and Duby (2000a). This cultural shift also influenced perceptions of illness and death, promoting a more personal and subjective experience of dying (Ariès 1999, 2005).

While the Order did not articulate a modern notion of autonomy, it contributed to the recognition of the subjectivity of the sick within the constraints of early modern caregiving practices (Foucault 1990). This recognition was not rhetorical, but embedded in regulatory texts that mandated attentive listening, individualized assessment, and moral accountability in care. Saint John of God emphasized the importance of personalized attention, as codified in the *Rule and Constitutions*:

Ask each one whether they are in need of anything, if they desire a particular comfort, or if they are being mistreated by caregivers who fail to provide what is necessary, so that these matters may be discreetly and prudently resolved.

(Méndez de Salvatierra 1585, Title VII, Const. 10)

This directive was reaffirmed in the Constitution of 1611: “Whatever is done to any of the poor or sick is received by Jesus Christ our Redeemer as if it were done unto His own person” (Hospitaller Order of Saint John of God, 1611, art. 74).

This principle was also passed on to future caregivers:

Then he shall wash his feet, and kiss them with all charity, remembering that our Father Saint John of God, in attempting to do this, found himself not before a poor man, but before Christ Himself; and not before a repugnant foot, but one that, though wounded, shone with splendor.

(Victoria 1668, chap. XXIII, § II)

These texts articulate a theological anthropology in which care is sacramentalized: the body of the sick becomes a locus of divine presence and ethical responsibility. Far from remaining symbolic, these precepts were ritually enacted and normatively preserved, forming the basis for practices that anticipated key dimensions of modern humanized care. Indeed, many of the Order's ethical principles—such as the duty to accompany the dying, the right to dignified treatment regardless of status, and the emphasis on an ethical code—remain foundational in contemporary healthcare ethics.

### 3.2 The Conception of Death in the Hospitaller Order of Saint John of God

In late medieval and early modern Catholic thought, death was conceived as a spiritual struggle between celestial and demonic forces, wherein the soul had to resist final temptations to attain salvation. The term *“death”* was laden with rhetorical and symbolic meaning, producing a semantic dualism between physical extinction— *“the first death”*—and spiritual condemnation through sin— *“the second death”*—the latter being the most feared (Mitre 2019). This worldview shaped a shared imaginary, visible in artistic motifs such as the *Danse Macabre* and in spiritual manuals like the *Ars Moriendi*, which sought not only to guide the dying but to structure the rituals, gestures, and prayers of end‐of‐life care (Martín 2004; Ariès 2005; Haindl Ugarte 2013). Within this theological framework, death was faced with fear and uncertainty, as one's eternal fate was seen to depend on an invisible spiritual battle beyond human control.

However, a shift in this paradigm emerged in the sixteenth century, driven by the influence of Christian humanism (Fernández Álvarez 1984; Huizinga 2014). Death began to be reinterpreted not merely as divine punishment, but increasingly as a hopeful transition toward salvation, reinforcing the need for ongoing interior conversion and structured spiritual vigilance (Martínez Gil 1993; Ariès 2005; Haindl Ugarte 2013). Under the impact of the Protestant Reformation and the Council of Trent (1545–1563), the Hospitaller Order of Saint John of God institutionally incorporated this evolving theological perspective. While not introducing a radically new vision, the Order strengthened the understanding of death as a release from suffering and an entry into eternal life (Iglesia Católica 1926; Ventosa 2012), anchored in trust in divine mercy (Amezcua 2019). This theological shift would shape not only doctrine but also concrete caregiving protocols oriented toward spiritual readiness.

This conception of death entailed the need for constant spiritual readiness, as salvation was believed to depend on the state of the soul at the moment of death. Within the Order, this belief translated into a systematic approach to care that affirmed both the physical and spiritual dimensions of the person, reflecting an integrative anthropological vision. The practice of accompanying the dying reveals a move from an Augustinian framework toward an Aristotelian‐Thomistic model, one that upheld the essential unity of body and soul (Papini 1954; Copleston 1960; Royo Marín 1963; Mitre 2019). This synthesis is also visible in the recorded death of Saint John of God himself, who, before dying, pronounced “Jesus, into your hands I commend my spirit” (Martínez Gil 2006, 410), and peacefully surrendered his soul to God at the age of 55.

For the Order, dying well required living well. As Saint John of God admonished, “Since the Lord will judge us as He finds us, it is wise to reform ourselves in time, and not be like those who say ‘tomorrow’ and ‘tomorrow’—and never begin” (Second Letter to the Duchess of Sessa, Fernández de Viana y Vieites et al. 2006, 92–93). This emphasis on progressive spiritual growth was not left to personal initiative alone but was institutionally embedded in the formation process. During novice training, the *Instruction* stated: “Examine the acts of virtue performed each day to see whether they were more perfect than the day before, for on the path of virtue, failing to advance is to fall behind” (Victoria 1668, chap. XIII, 61).

These maxims functioned as regulative principles of care, shaping the moral disposition of caregivers and reinforcing the Order's pedagogical model of continuous readiness for death.

Given that the precise hour of death was unknown, the brothers extended this call to preparation from the moment a patient entered the hospital. This vigilance was not occasional, but constituted a permanent spiritual framework integrated into the daily rhythm of care. The sick were accompanied with spiritual exercises, doctrinal instruction, and daily guidance, ensuring that care of the soul did not depend solely on deathbed rituals (Ventosa 2012). While the brothers practised this discipline with greater rigor, the poor and sick were likewise encouraged to cultivate their spiritual health throughout their stay, in anticipation of a sudden or unprepared death.

One powerful means of nurturing this spiritual readiness was the contemplation of Christ's Passion, which offered strength, consolation, and eschatological meaning in suffering (Kempis 2014). This practice was not merely personal, but systematically encouraged and ritualized as part of the care process: “To gaze upon and contemplate Jesus Christ crucified, and to reflect on his most holy Passion and the sufferings he endured in this life—all for us sinners” (Fernández de Viana y Vieites et al. 2006, 91).

You, Lord, through these most holy sufferings that you endured—and that I, a wretched sinner, now bring to mind—and through your holy cross and death, deliver me […] and grant that I may go where you took the good thief, crucified at your side.

(Victoria 1668, chap. XXVI, 156)

In this spiritual horizon, Christ's death was the ultimate act of self‐giving for humanity. Its ritual evocation during moments of agony endowed the final passage with redemptive meaning and grounded the patient's trust in divine compassion. These prayers were not improvised but institutionally transmitted and liturgically guided, forming part of a communal process of spiritual accompaniment. A contemporary witness to Saint John of God affirmed: “You go safely and need not fear […] your sins are forgiven through my death and precious blood, and through the works of mercy you have performed” (Martínez Gil 2006, 448).

In this way, the Order's conception of death transformed the experience of dying into a process of peaceful surrender rather than anguished struggle. Its model of care, prioritizing the most destitute, combined spiritual preparation with the alleviation of physical and emotional suffering.

While the Order of Saint John of God was not the only hospitaller institution of the seventeenth century, it was the first to receive papal recognition and to codify its caregiving ethos into binding regulations. These formalized principles became reference points for other Catholic hospital initiatives that emerged in the late sixteenth century and matured in the seventeenth, contributing to the development of a distinct model of spiritualized and humanized care (Amezcua 2015, 2019). Ultimately, these principles were not left to individual discretion or charismatic inspiration, but were institutionally systematized, enforced through training, and embedded in daily practice.

### 3.3 Care for the Dying in the Order of Saint John of God

Reflecting the Order's vision of death as a hopeful transition, the Hospitaller brothers developed explicit and normatively codified practices for caring for patients at the end of life. Since agony was understood as the threshold of individual judgment (Ariès 2005), the brothers ensured that no one faced this moment alone. This accompaniment was not left to spontaneous goodwill but constituted a formal and mandatory act of spiritual care embedded in the Order's mission.

From its beginnings, the Order emphasized the accompaniment of the dying—a principle that evolved from personal commitment into a legally binding institutional obligation. This process of formalization was initiated by the papal bull *Licet ex debito* (1572), which conferred official recognition and doctrinal legitimacy to the mission of the Order (San Pío 1572). It was then progressively codified in the *Constitutions of the Hospital of John of God* (1585) and the *General Constitutions* of 1587, which mandated that all patients in danger of death receive structured spiritual support. The 1611 Constitution marked the full institutionalization of this duty:

When a patient is in danger of death or at the point of death, the chaplain or a brother must remain constantly at their side, guiding them toward the salvation of their soul and offering them help and encouragement with some sustenance to comfort them.

(Hospitaller Order of Saint John of God, 1611, art. 77)

This regulation established spiritual accompaniment as an institutional duty and reaffirmed the central role of nursing care for the dying (Gómez Bueno 1963).

In the formation of novices, end‐of‐life care was described as a sacred and formative duty. Brothers were explicitly instructed to accompany the dying with gentleness and spiritual vigilance: “Encourage the patient to repeat many times the sweet name of Jesus […] exhorting gently, so as not to tire him” (Victoria 1668, chap. XXVI, 145). This practice was not only taught but also modeled by Saint John of God himself. According to tradition, during his final days, he was surrounded by disciples and friends who prayed and accompanied him until his death (Martínez Gil 2006). In this way, care for the dying was both a doctrinal precept and a pedagogical practice, ensuring its continuity through ritualized learning and lived example.

The institutionalization of end‐of‐life care ensured the preservation of patient dignity and formed part of a broader process of ritual regulation and spiritual discipline in the act of dying. From a Foucauldian perspective, this model may be understood as a disciplinary space in which death ceased to be an entirely domestic or private event, becoming a moment ritually organized, spiritually framed, and medically overseen by the institution (Foucault 1990, 2002, 2004). Although the Order promoted compassionate and personalized care, it also contributed to the normalization of death, with the hospital functioning simultaneously as a space of healing, spiritual assistance, and moral surveillance. This reading aligns with broader historiographical debates on early modern institutions as agents of emotional regulation and social discipline, shaping bodies, feelings, and attitudes toward illness, dying, and memory (Elías 1987; Foucault 2002).

Ultimately, the Order's approach to end‐of‐life care—as established in the Constitutions of 1587 and 1611—was gradually institutionalized and replicated throughout its hospitals, becoming a transnational model of structured spiritual care. This marked a significant departure from medieval representations of agony as a time of terror and eschatological uncertainty. The Saint John of God model promoted a peaceful, ritualized attitude toward death, free from superstition and despair (Martínez Gil 1993), and was fully consistent with Norbert Elias's (1987) concept of the *civilizing process*, which sought a balance between emotional expression and social regulation. While encouraging manifestations of faith and trust in divine mercy, the Order also discouraged excessive displays of fear or emotional breakdown that might disrupt the serenity of the dying person or the collective ethos of the hospital environment.

#### 3.3.1 The Sacraments of Penance, Communion, and Extreme Unction as Spiritual Assistance in Dying

Hospitaller care systematically guaranteed the administration of the sacraments—understood as essential for eternal salvation—and made it a nonnegotiable institutional priority that no patient should die without receiving them. As stated in the 1611 Constitution: “Take particular care and exercise great diligence to ensure that the sacraments are administered to the sick, so that no one dies without them” (Constitution of the Hospitaller Order of Saint John of God, 1611, art. 76).

This obligation had already been articulated in the 1585 *Rule and Constitutions*, which exhorted the brothers to remain at the patient's side until the final moment: “If the illness worsens, they shall do all they can, remaining with the patient during that harsh and critical moment of death, helping them to die well” (Méndez de Salvatierra 1585, Title IX, Const. 5).

The *Manual for the Administration of Sacraments* (1666) systematized the theology and pastoral function of Extreme Unction, listing its effects as: “to increase grace, forgive venial sins, remove the remnants of mortal sin, provide strength against the devil's temptations, and restore physical health if appropriate” (Orden Hospitalaria de San Juan de Dios 1666, chap. I, § V, 33). In this framework, the sacraments were not symbolic gestures but indispensable rituals of spiritual protection and salvation, essential to ensure a dignified and prepared death (Ariès and Duby 2000b).

Hospitaller caregivers were also responsible for maintaining uninterrupted spiritual vigilance over the dying, as solitude at the deathbed was perceived not only as neglect but as a grave failure of pastoral and theological duty (Ariès 2005). Once a patient entered the final stage, the administration of the sacraments intensified: the *Viaticum* (Eucharistic communion) and Extreme Unction were offered to ensure the soul's passage in a state of grace.

The ritual administration of the triad of Confession, Communion, and Unction—the so‐called *dormientium exitium* or “sacrament of the death of sleepers”—became a core liturgical practice. It provided not only emotional peace, but the theological assurance of salvation, reinforcing the idea that death, while feared, could be embraced as a sacramentally mediated threshold of eternal life (Ariès and Duby 2000a; Ariès 2005).

#### 3.3.2 The Dying Environment and the Role of Prayer

Prayer, a foundational element of Christian life since the Rule of Saint Benedict (fourth century) and further institutionalized by the Fourth Lateran Council (thirteenth century), was central to the dying process in Hospitaller care (Ariès and Duby 2000a). It created an atmosphere of faith, hope, and spiritual containment, providing both theological reassurance and physiological calm, as some studies have shown that prayer reduces agitation and promotes autonomic balance at the end of life (Bernardi et al. 2001).

Communal prayers included the Psalm Miserere, the Credo, and the Litany of the Saints, all imploring divine mercy for the soul of the dying (Méndez de Salvatierra 1585). Over time, the recitation of the Credo became a standardized element of the dying process, functioning as a devotional script akin to the Ars Moriendi, but adapted to the specific needs and vulnerabilities of terminal patients (Orden Hospitalaria de San Juan de Dios 1666).

The dying environment was meticulously arranged with symbolic and sacramental elements designed to strengthen the patient's faith and promote a peaceful spiritual transition. A crucifix was placed in their hands as a reminder of Christ's Passion, and lit candles symbolized divine light guiding the soul toward eternity (Orden Hospitalaria de San Juan de Dios 1587, chap. 20). Specific instructions emphasized ritual precision: “As soon as the patient is recognized as approaching death, the person assisting them shall help them to die well, placing a crucifix nearby, with a lit candle and holy water” (Victoria 1668, chap. XVI, 144–145).

Likewise, the *Manual for the Administration of Sacraments* outlined the spatial preparation required: “Prepare in the cell or infirmary (if there is no altar) a table draped with clean cloths, and place on it a devotional image or cross” (Orden Hospitalaria de San Juan de Dios 1666, chap. I, § III, 14–15). Through these elements, the deathbed became a ritualized sacred space, mediating between the physical realm and the eschatological horizon.

Through their continuous and compassionate presence, the Hospitaller brothers fostered a sacred space where the dying could express their fears, hopes, and final desires in the form of prayer, confession, or simple silence. This closeness cultivated spiritual intimacy and enabled a profound encounter between the individual and the divine (Ariès and Duby 2000b).

Far from being merely ritual assistants, the brothers acted as spiritual witnesses, accompanying each patient's final moments with a blend of empathy, theological understanding, and emotional availability. In doing so, they embodied a model of care that integrated doctrine, ritual, and human connection—an approach that remains deeply relevant to contemporary nursing ethics and end‐of‐life care.

#### 3.3.3 A Holistic Approach to Dying Care

During the final stage of life, the Hospitaller Order integrated the alleviation of physical suffering and emotional distress into its comprehensive caregiving model. This approach was not left to personal discretion but formally codified in the 1611 Constitution, which explicitly stated: “The brothers must provide sustenance—food or drink—to strengthen and comfort the dying” (Constitution of the Hospitaller Order of Saint John of God, 1611, art. 77). In this way, material care was seen as a concrete expression of spiritual compassion, and physical comfort was understood not as an end in itself, but as a preparation for the soul's final journey.

The *Instruction for Novices* provided detailed guidance for moments of extreme physical decline, highlighting the ethical sensitivity required in acts of bodily care: “If the patient is so incapacitated that he cannot or will not open his mouth to receive sustenance, gently open it (taking care not to harm him) with a spoon or knife and feed him” (Victoria 1668, chap. XXIII, § II, 118).

This instruction exemplifies the radical intimacy and embodied compassion that defined Hospitaller caregiving. Feeding the dying was not a mere technical gesture, but a ritualized act of tenderness, embodying the brother's role as a servant of both body and soul. In such moments, material assistance became a form of spiritual hospitality, reinforcing the patient's dignity through loving presence and tactile devotion.

Physical care was inseparable from spiritual consolation. As instructed in the *Instruction for Novices*, the dying were not only comforted, but ministered to through gestures of sacred intimacy: “Then wash his feet and kiss them with complete charity… undress him, lay him down, and encourage him with the hope of recovery, and even more, exhort him to embrace God's will” (Victoria 1668, chap. XXIII, § II, 115).

These actions—rooted in the symbolism of the Last Supper and Christ's washing of the feet—transformed routine tasks into acts of theological embodiment. They reinforced the idea that to care for the sick was to serve Christ himself, and that dying was not a medical failure, but a spiritually meaningful journey. The brother thus became both caregiver and catechist, guiding the body and the soul into final surrender.

The *Manual for the Administration of Sacraments* reminded caregivers that “the sick in hospitals need spiritual medicine no less than physical medicine” (Orden Hospitalaria de San Juan de Dios 1666, chap. I, § I, 6). This affirmation encapsulates the foundational ethos of Hospitaller care: the inseparability of body and soul, and the need to attend to both with equal reverence (Fernandes de Freitas and Siles González 2008).

In this perspective, death was not approached as a clinical endpoint, but as a sacred passage, and caregiving aimed to provide a peaceful, dignified, and spiritually integrated transition. The Hospitaller model thus anticipated contemporary calls for humanized end‐of‐life care, where dignity, presence, and compassion are central (Watson 2012).

### 3.4 Post‐Mortem Care in the Order of Saint John of God

The post‐mortem practices of the Hospitaller Order of Saint John of God, consolidated throughout the sixteenth and seventeenth centuries, were structured around four core dimensions: reverence for the deceased body, the solemnity of funeral rites, intercessory prayer for the soul, and ethical management of the patient's remaining assets. These practices were not spontaneous expressions of piety, but normatively codified procedures that reflected a coherent theology of death and a communal ethic of remembrance. In doing so, the Order extended its care beyond the moment of death, integrating physical, spiritual, and institutional responses to human finitude.

#### 3.4.1 Respect for the Body of the Deceased

From its earliest regulatory texts, the Hospitaller Order of Saint John of God emphasized the obligation to treat the deceased's body with reverence and ritual decorum. The *Rule and Constitutions* of 1585 explicitly mandated that “after death, the bodies shall be shrouded with dignity” (Méndez de Salvatierra 1585, Title IX, Const. 6). Similarly, the *General Constitutions* of 1587 stipulated that “having expired and surrendered the soul to God, the body shall shortly be shrouded and removed from the infirmary” (*Order of Saint John of God*, 1587, Title I, chap. 21).

The Order's post‐mortem protocol prescribed placing the deceased in a composed position, with the eyes closed and the hands crossed over the chest. As stated in the *Instruction for Novices*:

The nurse shall arrange the deceased's body according to established custom, dressing it with robe, belt, and scapular […] placing the hands crossed over the chest with a small cross between them, and laying the body and face uncovered in an open coffin.

(Victoria 1668, chap. II, § II, 82)

The deceased were typically shrouded in white linen made from old hospital sheets or other donated cloths, reflecting the Order's ethos of humility and simplicity: “using white linen from old hospital sheets, or any other cloth donated for that purpose” (Méndez de Salvatierra 1585, Title IX, Const. 6).

The 1611 Constitution reinforced these post‐mortem practices with additional ritual elements that sacralized the liminal space between death and burial. It prescribed that “after the patient has expired, the body shall remain on the bed for a while before being taken to the preparation room. During this time, light and holy water must not be absent” (Victoria 1668, chap. XXIII, 122).

Moreover, the *Manual for the Administration of Sacraments* explicitly warned against premature burials, emphasizing the need for prudence and reverence in the treatment of the deceased: “when death occurs suddenly, burial should not take place until twenty‐four hours have passed, due to the risk of apparent death” (Order of Saint John of God, 1666, chap. III, § I).

This respect for the deceased extended beyond ritual obligation or hygienic concern; it was grounded in a coherent anthropological and theological framework. Rejecting a radical dualism between body and soul, the Order adopted the Aristotelian‐Thomistic view of the human being as an integrated unity (*unitas substantialis*), in which the body retained ontological dignity even after death. Accordingly, the corpse was not seen as a discarded shell but as a meaningful expression of the human person, deserving of reverence and care (Copleston 1960; García Martínez 2014).

These rituals also served a broader social and anthropological function, contributing to what Norbert Elias (1987) termed the “civilizing process.” By standardizing and ritualizing post‐mortem care, the Order facilitated the transformation of instinctive emotional responses—such as fear, sorrow, or revulsion—into structured gestures of reverence, compassion, and hope. In this way, care for the dead aligned not only with hygienic imperatives but also with the institutional enactment of humility, spiritual continuity, and dignity.

#### 3.4.2 Funeral Rites and Communal Accompaniment

The funeral was conceived not merely as an individual transition but as a communal rite that extended spiritual care beyond death. In the ritual framework of the Order, the deceased was never abandoned; their memory and dignity were affirmed through liturgical presence and collective prayer. The *Manual for the Administration of Sacraments* established a normative sequence of rites:

as soon as the patient dies, the brothers shall rise and chant the Responso Subvenite, and the sacristan shall signal the death with the bells […] The Prayer of the Holy Shroud shall also be recited for the soul of the deceased.

(Order of Saint John of God, 1666, chap. II, § II)

The importance of these rites was such that they were formally integrated into the training of novices. As stipulated in the *Manual for the Administration of Sacraments*, “Let them learn the Psalms and Responsories required for the burial of the poor, along with the relevant devotions and prayers” (Order of Saint John of God, 1666, chap. V, 23). This institutional requirement ensured not only liturgical competence, but also the transmission of a collective ethic of remembrance and reverence.

After death, the body was transferred to the hospital chapel or church for the celebration of the requiem mass, a ritual that embodied both ecclesial solemnity and community participation. The *Rule and Constitutions* detailed this collective obligation:

The body shall be placed on a bier in the nave of the church, covered with a black cloth kept by the sacristan. All brothers present in the hospital, and any other available persons, must attend the funeral. Those who, without valid reason, fail to attend shall forfeit their daily rations—receiving only bread. Any brother who neglects this duty shall receive only bread and water.

(Méndez de Salvatierra 1585, Title IX, Const. 6)

These rites were intended to intercede for the soul of the deceased, reflecting the theological belief in the efficacy of prayer to alleviate post‐mortem suffering. One of the prescribed prayers reads:

My Lord Jesus Christ… I humbly implore you that this same charity […] be offered for the soul of this your servant […] and deliver him, Lord, from all tribulations and pains he may fear to have merited through his sins.

(Order of Saint John of God, 1666, chap. XXVI, 158–159)

This liturgical formula demonstrates the Order's spiritual anthropology, in which mercy, intercession, and collective prayer were essential to ensuring a dignified passage into eternity.

Unlike many hospitals of the period—where resource scarcity frequently led to anonymous and unceremonious mass burials—the Order of Saint John of God established institutional norms to ensure that even the most destitute received a Christian burial. In hospitals that maintained their own cemeteries, the deceased were interred in consecrated ground, reinforcing the Order's commitment to dignity beyond death and the theological importance of resting in sacred space (Martínez Gil 2006).

#### 3.4.3 Remembrance of the Deceased and Spiritual Intercession

The Order's commitment to the deceased extended beyond the moment of burial, incorporating structured remembrance and intercessory prayer as part of its institutional routine. Weekly requiem masses were held in all its hospitals, as formally prescribed in the 1587 Constitution: “Every Monday of the year a requiem mass shall be said for the poor deceased in each of our Houses” (Order of Saint John of God, 1587, Title I, chap. 22, 10).

This practice was reaffirmed in the 1611 General Constitution, which prescribed specific liturgical obligations both in the church and in the cemetery for the benefit of deceased benefactors, impoverished patients, and members of the religious community: “Masses and prayers shall be offered each week for the souls of the poor, the benefactors, and the brothers of the Order” (Order of Saint John of God, 1611, art. 22, 154).

These practices reflected the widespread early modern belief in the efficacy of suffrages—prayers, masses, and acts of piety—as intercessory means capable of reducing the soul's duration in Purgatory (Ariès 1999; Le Goff 1989). Their doctrinal and pastoral importance is illustrated by the fact that “Pope Clement VIII granted plenary indulgences to those who recited the Prayer of the Holy Shroud with devotion and true contrition, for the benefit of souls in Purgatory” (Order of Saint John of God, 1666, chap. II, § II).

Beyond their spiritual efficacy, these rituals played a psychosocial role by supporting the grieving processes of relatives, caregivers, and the hospital community (Murphy 2011). Through the reinforcement of memory and communal belonging, they contributed to the containment and ritualization of mourning. This function is consistent with Norbert Elias's (1987) concept of the “civilizing process,” whereby emotional expressions are gradually subject to social codes that promote decorum, continuity, and emotional regulation in public and institutional settings.

The *Manual for the Administration of Sacraments* also codified a ritual calendar for commemorating the dead, thereby structuring remembrance within a liturgical and communal framework. It established the obligation to celebrate the funeral mass not only on the day of burial, but also on the third, seventh, ninth, and thirtieth days following death—intervals aligned with Catholic eschatological tradition and symbolic numerology:

On the day of burial, the designated mass and prayers shall be celebrated, even if several days have passed since death […] On the third, seventh, and thirtieth days, the funeral mass and appropriate prayers shall be said. On the ninth day, the same funeral mass shall be repeated for the deceased in whose name the office is held.

(Order of Saint John of God, 1666, chap. III, § V, 117)

#### 3.4.4 Asset Management and Its Communal Dimension

In some cases, patients who died in the hospital left behind donations, personal belongings, or wills intended to support the institution (García Martínez 2007). In a society characterized by scarcity and pronounced economic inequality, as documented by Fernández Álvarez (2006) and Sanz and Camañes (2012), the responsible management of these resources was not only a practical necessity but also a moral imperative rooted in the Order's ethos of accountability and communal stewardship.

Aware of the potential for misappropriation or ethical conflict, the Order established precise regulatory mechanisms to ensure transparency and institutional integrity. According to the 1585 *Rule and Constitutions*:

There shall be an archive in the hospital with three keys […] where all money collected shall be deposited […] including funds found in possession of deceased patients who died intestate, proceeds from the auction of their garments, and all other assets rightfully belonging to the hospital.

(Méndez de Salvatierra 1585, Title XIV, Const. 3)

These resources were allocated to the maintenance of the hospital, the care of the poor and the sick, and the celebration of masses for the deceased. To guarantee accountability, meticulous record‐keeping was mandated (Del Río and Palma 2007). The 1611 *General Constitutions* specified: “money, bread, wine, oil, and all other items shall be delivered to the brothers responsible for their distribution, and recorded in the main ledger with day, month, year, and specific manner of use” (Order of Saint John of God, 1611, art. 103).

This meticulous approach not only ensured material accountability but also reinforced the Hospitaller brothers’ vow of poverty, promoting coherence between spiritual ideals and everyday practice. Economically, ethically, and symbolically, these post‐mortem practices embodied the Order's holistic vision of care—one in which material stewardship, liturgical intercession, and collective remembrance converged into an integrated expression of hospitality, charity, and eschatological hope.

## 4 Conclusion

Grounded in a qualitative and hermeneutic approach within the historiography of nursing, this study confirms the historical relevance of the Hospitaller Order of Saint John of God in sixteenth‐ and seventeenth‐century Spain. It underscores the Order's pivotal contribution to the development of early holistic models of hospital care, particularly in relation to structured end‐of‐life practices. Rooted in a consistent ethical and theological framework, the Order provided care that transcended the merely clinical, integrating spiritual, emotional, and physical dimensions up to the final moment of the patient's life.

The Order's conception of death as a hopeful transition toward salvation must be interpreted within the broader cultural transformation described by historians such as Ariès and Martínez Gil, who have shown how the late medieval focus on judgment and fear gradually gave way to a more pastoral and mercy‐centered vision of dying. This evolution—already noted by Ariès and Martínez Gil—did not eliminate eschatological fear but reframed it within a more pastoral and sacramentally hopeful horizon. Rather than framing death as a moment of anguish or eschatological uncertainty, the Hospitaller sources present it as a time for spiritual peace, sacramental presence, and communal intercession. Liturgical texts and normative protocols codified a structured, compassionate, and theologically coherent process: from the administration of the last rites to post‐mortem rituals and financial stewardship. These practices did not simply reflect the religious ideals of the time, but actively shaped a distinctive ethic of dying—one in which the hospital became not only a place of healing, but a sacred space of transition.

This perspective generated caregiving practices centered on continuous spiritual presence, sacramental administration, the creation of a ritually meaningful environment, and careful attention to the physical and emotional needs of the dying. Post‐mortem protocols—including care for the body, funeral rituals, and prayers for the dead—were normatively structured, revealing an ethics of care that extended beyond death itself. Similarly, the regulated management of the deceased's assets reflected institutional priorities of transparency, stewardship, and communal responsibility.

While the actions of the Order were firmly rooted in the theological and cultural paradigms of early modern Catholicism, they anticipated principles that resonate with contemporary bioethics, such as respect for patient dignity, integrative care for suffering, and the ethical treatment of the body after death.

Through a hermeneutic lens, this historical analysis provides a nuanced reconstruction of how the Hospitaller Order of Saint John of God contributed to shaping a more dignified, compassionate, and socially regulated experience of dying. By interpreting the Order's caregiving practices within their original symbolic and theological frameworks—while deliberately avoiding anachronistic projections—this study maintains historical fidelity and illuminates their enduring ethical and anthropological significance. In this way, the legacy of the Order transcends its early modern context and continues to offer valuable insight into the ongoing dialog between care, spirituality, and human vulnerability.

## Ethics Statement

This is a historical study based on archival and published sources. It does not involve human participants or animal subjects.

## Conflicts of Interest

The authors declare no conflicts of interest.

## Permission to Reproduce Material From Other Sources

All sources used in this article are in the public domain or cited according to standard academic practice. No additional permissions were required.

## Data Availability

This is a historical study based on archival sources and published literature. No datasets were generated or analyzed for this article.

## References

[nin70042-bib-0001] Agustín de Hipona . s.f. *Regla de San Agustín [Rule of Saint Augustine]*.

[nin70042-bib-0002] Amezcua, M. 2015. Los cuidados de enfermería en la Edad Moderna [Nursing Care in the Early Modern Age]. https://index-f.com/gomeres/?p=458.

[nin70042-bib-0003] Amezcua, M. 2019. Cuidados y sociedad en la España Moderna: Materiales para la historia de la enfermería en los siglos XVI–XVII [Care and Society in Early Modern Spain: Materials for the History of Nursing in the 16th–17th Centuries]. Fundación Index.

[nin70042-bib-0004] Antonelli, E. , I. De Renzi , and G. Pizzorusso . 2015. Historia de la Orden de San Camilo: La Provincia Española [History of the Order of St. Camillus: The Spanish Province]. Religiosos Camilos.

[nin70042-bib-0005] Ariès, P. 1999. El hombre ante la muerte [Man Before Death]. Taurus.

[nin70042-bib-0006] Ariès, P. 2005. Historia de la muerte en Occidente: Desde la Edad Media hasta nuestros días [The History of Death in the West: From the Middle Ages to the Present Day]. El Acantilado.

[nin70042-bib-0007] Ariès, P. , and G. Duby . 2000a. Historia de la vida privada: Tomo 2. De la Europa feudal al Renacimiento [History of Private Life: Vol. 2. From Feudal Europe to the Renaissance]. Taurus.

[nin70042-bib-0008] Ariès, P. , and G. Duby . 2000b. Historia de la vida privada: Tomo 3. Del Renacimiento a la Ilustración [History of Private Life: Vol. 3. From the Renaissance to the Enlightenment]. Taurus.

[nin70042-bib-0009] Bernardi, L. , P. Sleight , G. Bandinelli , et al. 2001. “Effect of Rosary Prayer and Yoga Mantras on Autonomic Cardiovascular Rhythms: Comparative Study.” BMJ 323, no. 7327: 1446–1449. 10.1136/bmj.323.7327.1446. 11751348 PMC61046

[nin70042-bib-0010] Cabañas, J. 2017. “La Espiritualidad En La Asistencia Hospitalaria Del Barroco [Spirituality in Hospital Care During the Baroque Period].” Revista de Historia Cultural y Religiosa 12, no. 2: 45–67.

[nin70042-bib-0011] Castro, F. 1995. Historia de la vida y santas obras de San Juan de Dios y de la Institución de su orden y principios de su Hospital [History of the Life and Holy Works of Saint John of God and the Foundation of His Order and Hospital]. Obra Cultural Cajasur.

[nin70042-bib-0012] Conejo da Pena, A. and Bridgewater Mateu, P. , eds. 2023. The Medieval and Early Modern Hospital: A Physical and Symbolic Space. Viella.

[nin70042-bib-0013] Copleston, F. C. 1960. El pensamiento de Santo Tomás [The Thought of Saint Thomas]. Fondo de Cultura Económica.

[nin70042-bib-0014] Díaz Villanueva, F. , and A. Garín . 2022. Lutero, Calvino y Trento: La Reforma que no fue [Luther, Calvin, and Trent: The Reformation That Wasn't]. Almuzara.

[nin70042-bib-0015] Elías, N. 1987. *El proceso de la civilización [The civilizing process]*. Fondo de Cultura Económica.

[nin70042-bib-0016] Fernandes de Freitas, G. , and J. Siles González . 2008. “Antropología y cuidados en el enfoque de San Juan de Dios.” Index de Enfermería 17, no. 2: 144–148. 10.6018/nie.288051.

[nin70042-bib-0017] Fernández Álvarez, M. 1984. La sociedad española en el Siglo de Oro [Spanish society in the Golden Age]. Editora Nacional.

[nin70042-bib-0018] Fernández Álvarez, M. 2006. Felipe II y su tiempo [Philip II and His Time]. Espasa.

[nin70042-bib-0019] Fernández Álvarez, M. 2015. Carlos V, el César y el Hombre [Charles V, the Caesar and the Man]. Espasa Calpe.

[nin70042-bib-0020] Fernández de Viana y Vieites, J. I. , M. T. González Balasch , M. Mina y Salvador , C. A. Plumed Moreno , and F. de la Torre Rodríguez . 2006. Cartas de San Juan de Dios [Letters of Saint John of God]. Fundación Juan Ciudad. https://www.ohsjd.org/Resource/CARTASSANJUANDEDIOSOK.pdf.

[nin70042-bib-0021] Foucault, M. 1990. Tecnologías del yo y otros textos afines [Technologies of the Self and Related Texts]. Paidós.

[nin70042-bib-0022] Foucault, M. 2002. Vigilar y castigar: Nacimiento de la prisión [Discipline and Punish: The Birth of the Prison]. Siglo XXI Editores Argentina.

[nin70042-bib-0023] Foucault, M. 2004. El nacimiento de la clínica: Una arqueología de la mirada médica [The Birth of the Clinic: An Archaeology of Medical Perception]. Siglo XXI Editores Argentina.

[nin70042-bib-0024] García Martínez, A. C. 2014. “Las constituciones de los hospitales y los cuidados enfermeros en la España de los Austrias (siglos xvi–xvii) [Hospital constitutions and nursing care in Habsburg Spain (16th–17th centuries)].” Erebea. Revista de Humanidades y Ciencias Sociales 4. 10.33776/erebea.v0i4.2497.

[nin70042-bib-0025] García Martínez, M. 2007. “Cuidar el cuerpo y salvar las almas: La práctica de la enfermería según el modelo de la Congregación de enfermeros obregones [Caring for the Body and Saving Souls: Nursing Practice in the Obregonian Model].” Thesis doctoral, Universidad de Sevilla, Repositorio institucional. http://hdl.handle.net/11441/15076.

[nin70042-bib-0026] García Oro, J. , and M. Portela Silva . 2000. “Felipe II y el problema hospitalario: Reforma y patronato [Philip II and the Hospital Problem: Reform and Patronage].” Cuadernos de Historia Moderna 25: 87–124. https://revistas.ucm.es/index.php/CHMO/article/view/CHMO0000220087A.

[nin70042-bib-0027] García‐Villoslada, R. 1979. Historia de la Iglesia en España: La Iglesia en la España de los siglos XV y XVI [History of the Church in Spain: The Church in 15th and 16th Century Spain]. Biblioteca de Autores Cristianos.

[nin70042-bib-0028] Gijón Jiménez, V. 2018. “Los viajeros extranjeros y los hospitales españoles de la última década del siglo XV hasta la Revolución Francesa [Foreign Travelers and Spanish Hospitals From the Late 15th Century to the French Revolution].” Revista Vectores de Investigación 12–13: 12–13.

[nin70042-bib-0029] Le Goff, J. 1989. El nacimiento del Purgatorio [The Birth of Purgatory]. Taurus.

[nin70042-bib-0030] Gómez Bueno, J. 1963. Historia de la Orden Hospitalaria de San Juan de Dios [History of the Hospitaller Order of Saint John of God]. Archivo Interprovincial Casa del Tránsito.

[nin70042-bib-0031] Granjel, L. 1980. La medicina española renacentista [Renaissance Spanish medicine]. Ediciones Universidad de Salamanca.

[nin70042-bib-0032] Haindl Ugarte, A. 2013. “Ars bene moriendi: El arte de la buena muerte [Ars Bene Moriendi: The Art of Dying Well].” Revista Chilena de Estudios Medievales 3: 89–108.

[nin70042-bib-0033] Hernández Martín, F. C. 1996. Historia de la enfermería en España: Desde la Antigüedad hasta nuestros días [History of Nursing in Spain: From Antiquity to the Present]. Síntesis.

[nin70042-bib-0034] Huizinga, J. 2014. Erasmo [Erasmus]. Ediciones Ulises.

[nin70042-bib-0035] Iáñez, E. 1989. Historia literatura 4: Las literaturas en el siglo XVII [History of Literature 4: Literatures in the 17th Century]. Bosch Casa Editorial.

[nin70042-bib-0036] Iglesia Católica . 1926. Catecismo romano promulgado por el Concilio de Trento [Roman Catechism Promulgated by the Council of Trent], edited by T. A. M. Gubianas . Editorial Litúrgica Española. https://adelantelafe.com/wp-content/uploads/2015/12/CATECISMO-ROMANO-CONCILIO-DE-TRENTO-520p.pdf.

[nin70042-bib-0037] Kempis, T. 2014. Imitación de Cristo [The imitation of Christ]. Edibesa.

[nin70042-bib-0038] León Vegas, M. 2013. “La ʻProtección Socialʼ En La Edad Moderna: Cofradías Y Fundaciones Pías En El Sur Peninsular (Siglo Xvi) [ʻSocial Protectionʼ in the Early Modern Age: Brotherhoods and Pious Foundations in Southern Spain (16th Century)].” Baetica. Estudios de Arte, Geografía e Historia 35: 283–297.

[nin70042-bib-0039] López Terrada, M. L. , and C. Schmitz . 2018. “Licencias sociales para sanar: la construcción como expertos de salud de curanderas y curanderos en la Castilla del barroco.” Studia Historica: Historia Moderna 40, no. 2: 143–175. 10.14201/shhmo2018402143175.

[nin70042-bib-0040] Martín, J. L. 2004. Historia de España: Plena y Baja Edad Media. De la Reconquista a la expansión atlántica (siglos XI–XV) [History of Spain: High and Late Middle Ages. From the Reconquest to Atlantic expansion (11th–15th centuries)]. Espasa Calpe.

[nin70042-bib-0041] Martínez Gil, F. 1993. Muerte y sociedad en la España de los Austrias [Death and Society in Habsburg Spain]. Siglo XXI de España.

[nin70042-bib-0042] Martínez Gil, J. 2006. Proceso de beatificación de San Juan de Dios [Beatification Process of Saint John of God]. Biblioteca de Autores Cristianos.

[nin70042-bib-0043] Méndez de Salvatierra, J. 1585. Regla y Constituciones para el Hospital de Juan de Dios de la ciudad de Granada [Rule and Constitutions for the Hospital of Saint John of God in Granada]. En casa de Hugo de Mena.

[nin70042-bib-0044] Mitre, E. 2019. Morir en la Edad Media: Los hechos y los sentimientos [Dying in the Middle Ages: Facts and Feelings]. Ediciones Cátedra.

[nin70042-bib-0045] Murphy, N. 2011. “Immortality Versus Resurrection in the Christian Tradition.” Annals of the New York Academy of Sciences 1234, no. 1: 76–82. 10.1111/j.1749-6632.2011.06132.x. 21988252

[nin70042-bib-0046] Orden Hospitalaria de San Juan de Dios . 1587. Constituciones de la Orden Hospitalaria de San Juan de Dios [Constitutions of the Hospitaller Order of Saint John of God]. Roma.

[nin70042-bib-0047] Orden Hospitalaria de San Juan de Dios . 1666. *Manual de administración de sacramentos: Instrucciones para la correcta administración de los sacramentos a los enfermos [Manual for the Administration of Sacraments: Instructions for Properly Administering Sacraments to the Sick]*.

[nin70042-bib-0048] Orden Hospitalaria de San Juan de Dios . 2019. *Cronología histórica 1570–1700. Identidad e historia de la Orden Hospitalaria [Historical Chronology 1570–1700. Identity and History of the Hospitaller Order]*. https://escueladehospitalidadprovinciadecastilla.wordpress.com/cronologias-historicas/cronologia-historica-siglo-xviii/.

[nin70042-bib-0049] Orden Hospitalaria de San Juan de Dios . (s.f.). La Orden Hospitalaria de San Juan de Dios. Provincia de España [The Hospitaller Order of Saint John of God. Province of Spain] . https://sjd.es/wp-content/uploads/2022/01/Dossier-San-Juan-de-Dios-Provincia-de-Espana.pdf.

[nin70042-bib-0050] Papini, G. 1954. San Agustín [Saint Augustine]. Ediciones Fax.

[nin70042-bib-0051] Del Río, M. I. , and A. Palma . 2007. “Cuidados Paliativos: Historia y desarrollo [Palliative Care: History and Development].” Boletín Escuela de Medicina U.C. 32, no. 1: 5–12.

[nin70042-bib-0052] Royo Marín, A. 1963. Teología de la caridad [Theology of Charity]. Biblioteca de Autores Cristianos.

[nin70042-bib-0053] San Pío, V. 1572. *Bula Licet ex debito* [Papal Bull Licet ex Debito].

[nin70042-bib-0054] Sánchez‐Martínez, J. , ed. 2007. “Pleito entre los hermanos del ‘ospital de Juan de Dios’ y ‘los frayles e convento del monasterio de San Gerónimo’ [Dispute Between the Brothers of the Hospital of Saint John of God and the Monks of the Monastery of Saint Jerome].” In En *Hospital de San Juan de Dios: Construcción y propiedad histórica (1543–1593)* , 133–436. Archivo Museo San Juan de Dios.

[nin70042-bib-0055] Sanz , and P. Camañes . 2012. Atlas histórico de España en la Edad Moderna [Historical atlas of Spain in the Early Modern Age]. Síntesis.

[nin70042-bib-0056] Siles , and J. González . 2009. Historia de la enfermería [History of Nursing]. Editorial Aguaclara.

[nin70042-bib-0057] Ventosa, F. 1984. Historia de la Enfermería Española [History of Spanish Nursing]. Editorial Ciencia 3.

[nin70042-bib-0058] Ventosa, F. 2012. Pensamiento de San Juan de Dios y la Orden Hospitalaria y su relación con la Enfermería: Conceptos y valores [Thought of Saint John of God and the Hospitaller Order and Its Relationship With Nursing: Concepts and Values]. Archivo‐Museo San Juan de Dios “Casa de los Pisa”.

[nin70042-bib-0059] Victoria, A. de . 1668. *Instrucción de novicios de la Orden de la Hospitalidad de Nuestro Padre San Juan de Dios [Instruction for Novices of the Order of Hospitality of Our Father Saint John of God]*. Madrid.

[nin70042-bib-0060] Watson, J. 2012. Human Caring Science: A Theory of Nursing. 2nd ed. Jones & Bartlett Learning.

